# Serum IL-17 & eotaxin levels in asthmatic patients with allergic rhinitis

**DOI:** 10.12669/pjms.323.9914

**Published:** 2016

**Authors:** Hong Lv, Bing Lu, Xing-jia Qian, Jian-an Huang, Tie-feng Qiu

**Affiliations:** 1 Hong Lv, Department of Respiratory Medicine, Taicang Hospital of Traditional Chinese Medicine, Suzhou, Jiangsu 215400, P. R. China; 2 Bing Lu, Department of Respiratory Medicine, Taicang Hospital of Traditional Chinese Medicine, Suzhou, Jiangsu 215400, P. R. China; 3 Xing-jia Qian, Department of Respiratory Medicine, Taicang Hospital of Traditional Chinese Medicine, Suzhou, Jiangsu 215400, P. R. China; 4 Jian-an Huang, Department of Respiratory Disease & Critical Care Medicine, The First Affiliated Hospital of Soochow University, Suzhou, Jiangsu 215006, P. R. China; 5 Tie-feng Qiu, Department of Respiratory Medicine, Affiliated Wujin Hospital of Jiangsu University, Changzhou, Jiangsu 213002, P. R. China

**Keywords:** IL-17, Eotaxin, Asthma, Allergic rhinitis

## Abstract

**Objective:**

To investigate the serum levels of Interleukin (IL)-17 and eotaxin levels and the relationship between serum IL-17, eotaxin and pulmonary function in asthmatic patients with allergic rhinitis.

**Methods:**

Serum IL-17 and eotaxin levels in asthmatic patients with allergic rhinitis during attacking and remission and in healthy control subjects were measured using enzyme-linked immunosorbent assay (ELISA) kits. Then we studied the correlation between the serum IL-17, eotaxin levels and pulmonary function in patients.

**Results:**

Serum IL-17 and eotaxin levels were significantly elevated in patients during asthma attack and remission compared with healthy control subjects. These levels in patients during asthma attack were much higher than those during remission. Furthermore, serum IL-17 and eotaxin levels were negatively correlated with pulmonary function in asthmatic patients with allergic rhinitis, respectively.

**Conclusion:**

Our findings suggest that IL-17 and eotaxin are important factors in asthma with allergic rhinitis, and the correlation between serum IL-17, eotaxin and lung function possibly lead to improvements in the diagnosis and treatment of asthma with allergic rhinitis and related diseases.

## INTRODUCTION

Asthma is a chronic inflammatory disease of airways characterized by chronic inflammation, airway hyper-reactivity, and by symptoms of coughing, wheezing and chest tightness. Executive summary of the Global Initiative of Asthma (GINA) Dissemination Committee report in 2004 estimated that 300 million people of all ages, and all ethnic backgrounds, suffered from asthma, and there would be 400 million persons with asthma by 2025.^1^ Allergic rhinitis is an inflammatory nasal disorder characterized by one and more symptoms including sneezing, itching, nasal congestion, and rhinorrhea. Moreover, in previous epidemiological studies, rhinitis was found to occur in 80% of patients with asthma.^2^

Interleukin (IL)-17, a newly described cytokine secreted by activated subtype of T helper lymphocytes Th17,^3^ can induce the production of IL-6, prostaglandin E2, proliferation of T cells as well as growth and differentiation of CD34^+^ human progenitors into neutrophils.^4^,^5^ The increased expression of IL-17 was found to be associated with many inflammatory diseases including asthma, rheumatoid arthritis, systemic lupus erythematosus and multiple sclerosis.^6^-^9^ Recently, increasing evidence linking that IL-17 plays a critical role in allergic rhinitis and even related to the clinical severity in allergic rhinitis.^10^,^11^ Eotaxin is an eosinophil-specfic CC chemokine associated with the recruitment of eosinophils to the site of allergic inflammation. During asthma, mast cells and Th2 lymphocytes are activated in the lung to generate IL-4, IL-3 and tumour necrosis factor (TNF)-α.^12^ These cytokines stimulate the generation of eotaxin, which acting on CC chemokine receptor 3 (CCR3) on eosinophilsthen stimulate the migration of eosinphils into the lung tissue. Recent study reported that eotaxin could activate extracellular signal regulated kinase 2 (ERK2) and p38 mitogen activated protein kinase (MAPK) to stimulate the migration of eosinphils to epithelial cells, then activate, produce and release several inflammatory mediators to induce airway inflammation.^13^ In addition, the elevated eotaxin levels were also observed in allergicrhinitis.^14^ All these above suggested that IL-17 and eotaxin play a critical role in asthma and allergic rhinitis.

However, the pathophysiological role of IL-17 and eotaxin in asthmatic patients with allergic rhinitis are still unclear. To determine whether IL-17 and eotaxin play a role in asthma with allergic rhinitis, we explored the serum of IL-17 and eotaxin levels and the relationship between serum IL-17, eotaxin and disease severity as measured by FEV1%pred.

## METHODS

### Subjects

The study protocol was approved by the Ethics Committee of Taicang Hospital of Traditional Chinese Medicine, and informed written consents were obtained from all patients. Total twenty asthmatic patients with allergic rhinitis were enrolled in the study from the department of respiration of Taicang Hospital of Traditional Chinese Medicine, Suzhou, P. R. China. Diagnosis of asthma was performed according to the Chinese Guidelines on the Prevention and Management of Asthma. Diagnosis of allergic rhinitis was based on the allergic rhinitis diagnostic criteria established by the 2004 Otorhinolaryngology Society of the Chinese Medical Association (Lanzhou, 2005). All patients had no oral steroid intake or change of asthma or allergic rhinitis medications four weeks prior to recruitment of study. Twenty-three sex- and age-matched healthy non-allergic healthy volunteers were enrolled as controls.

### Samples

We collected blood samples from healthy subjects, patients during asthma attack and remission. Blood samples were drawn in serum tubes, clotted at room temperature for 10-20 minutes and centrifuged for 20 minutes at 3000 rpm. Serum samples were immediately frozen at -80 °C for further assay.

### Evaluation of pulmonary function

Immediately after the blood sampling, FEV1%pred was evaluated using a pneumotach meter system with Lily head (Master Screen Pneumo, Erich Jaeger, Germany).

### Measurement of serum IL-17 & Eotaxin levels

Serum IL-17 and eotaxin were measured by commercial enzyme-linked immunosorbent assay (ELISA) using reagent kits of Shanghai yifeng Biotechnology Co., Ltd. (Shanghai, P. R. China) according to the manufacturer’s instructions.

### Statistical Analysis

Data were analyzed by the SPSS 18.0 software (SPSS Inc., Chicago, IL, USA). Normally distributed data were expressed as the mean±SD and non-normally distributed data were expressed as the median (25-75th percentiles). The Student paired *t*-test was used to compare normally distributed data between two groups, and the rank sum test was applied for nonnormally distributed data between two groups. The χ^2^ test was used to compare frequencies. All reported p values were two-sided, and *p < 0.05* was considered statistically significant.

## RESULTS

### Characteristics of patients & healthy control subjects at inclusion

The demographics and clinical characteristics of the healthy control subjects and asthmatic patients with allergic rhinitis are shown in Table 1. No significant difference in age, sex was observed between patients and healthy control. As expected, patients with attacks showed lower FEV1%pred (mean±SD: 68.95±7.86 vs. 84.17±3.42, *p*< 0.05) compared to healthy control subjects (Table-I). However, the FEV1%pred levels were found higher in patients during remission than during asthma attack (*p*< 0.05). Eosinophils are the primary effector cells in the mechanism of asthma. As shown in Table-I, elevated levels of eosinophils (%) were also observed in patients with and without attacks in comparison to those in healthy control subjects (*p*< 0.05), the eosinophils (%) levels in patients with remission were greatly decreased than those during asthma attack (*p*< 0.05).

**Table-I:** Characteristics of patients and healthy control subjects.

	*Healthy control*	*Asthmatic patients with allergic rhinitis*	

		*Attacking stage*	*Remission stage*
Number	23	20	
Gender (Male/Female)	13/10	11/9	
Age (years)^b^	39.09±2.12	39.85±3.47	
FEV1%pred^b^	84.17±3.42	68.95±7.86^#^	81.95±3.76*
Eosinophils (%)^b^	2.24±0.19	6.67±1.01^#^	3.25±0.41*

b The data were expressed as the mean±SD.

# p< 0.05, compared with control group;

* p< 0.05, compared with attacking stage group.

### Serum levels of IL-17 & Eotaxin

**Fig.1:**
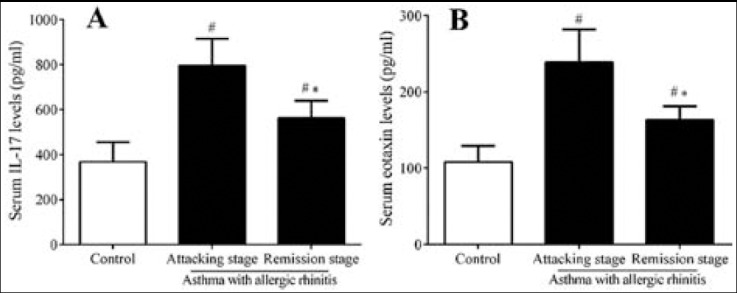
Serum levels of IL-17 and eotaxin in patients and healthy control subjects. The data were expressed as the mean±SD. #: p< 0.05, compared with control group; *: p< 0.05, compared with attacking stage group.

Serum IL-17 (Fig.1A) and eotaxin (Fig.1B) levels were significantly elevated in patients during asthma attack and remission (*p*< 0.05) compared with healthy control subjects. In addition, serum IL-17 and eotaxin levels were much higher during asthma attack than those during remission (*p*< 0.05).

### Relationship between pulmonary function, IL-17 & eotaxin

**Fig.2:**
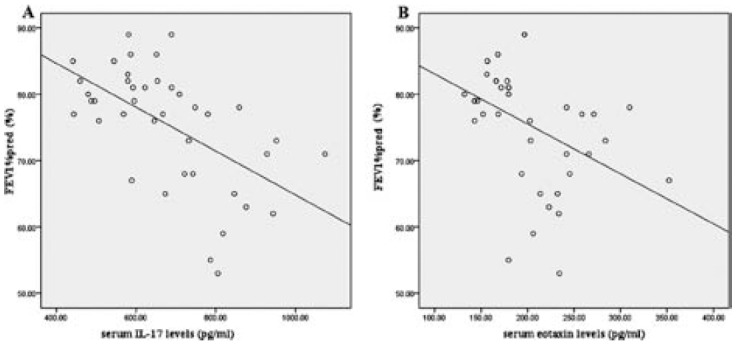
Correlation between pulmonary function, IL-17 and eotaxin in asthmatic patients with allergic rhinitis.

Pulmonary function tests were performed immediately after taking blood samples. As showed in Fig.2A, negative correlation was found between serum IL-17 levels and FEV1%pred in asthmatic patients with rhinitis (r = -0.570, *p*< 0.05). Furthermore, in patients the serum eotaxin levels significantly correlated with FEV1%pred (r = -0.422, *p*< 0.05, Fig. 2B).

## DISCUSSION

The present study showed significantly increased serum IL-17 and eotaxin levels in patients with and without asthma attacks compared to healthy subjects. Previous study had demonstrated that IL-17 levels were increased in sputum and bronchoalveolar lavage fluids (BALFs) of asthmatic patients.^15^ Recently, serum IL-17 levels were found higher in asthmatic patients than healthy subjects.^16^ IL-17 activated the release of eotaxin from airway smooth muscle cells, and also induced the release of inflammatory mediators from human eosinophils.^17^ Furthermore, an experimental study revealed that IL-17 is a negative regulator in the initiation of allergic asthma.^18^ Consistent with these, we demonstrated that elevated serum IL-17 levels were observed in patients during attacking, which were greatly decreased during remission g. Moreover, negative correlation was also found between serum IL-17 levels and FEV1%pred in asthmatic patients with allergic rhinitis. These results suggested that serum IL-17 levels play an important role and linked to the severity of asthma with rhinitis.

Similarly to IL-17, the meanserum levels of eotaxin increased in patients, increased transcription from eotaxin gene had been observed in the bronchial mucosa of asthmatic patients.^19^ Increased expression of eotaxin contributes to the chemotaxis of eosinophils to the site of inflammation.^20^ Our results showed that serum eotaxin levels had a negative correlation with pulmonary function in asthmatic patients with allergic rhinitis. This observation is consistent with previous report on the association of eotaxin and pulmonary function in asthmatic patients,^21^ which demonstrated that eotaxin levels contributed to the odds of the asthma diagnosis and of impaired pulmonary function.

In conclusion, this study showed that serum IL-17 and eotaxin levels were significantly elevated in asthmatic patients with allergic rhinitis during attacking, which were greatly decreased during remission, the serum IL-17 and eotaxin levels were negatively correlated with lung function in asthmatic patients with allergic rhinitis, respectively. Furthermore, serum levels of IL-17 and eotaxin are much easier measurable compared to these in sputum, BALFs or bronchial biopsies. Thus, the correlation between serum IL-17, eotaxin levels and lung function possibly leading to improvements in the diagnosis and treatment of asthma with allergic rhinitis and related diseases.

### Limitations of the Study

We evaluated a limited number of patients, and further work is also needed to clarify the precise mechanism of action of IL-17 and eotaxin in asthmatic patients with allergic rhinitis.
